# Catch Recognition in Automated American Football Training Using Machine Learning

**DOI:** 10.3390/s23020840

**Published:** 2023-01-11

**Authors:** Bernhard Hollaus, Bernhard Reiter, Jasper C. Volmer

**Affiliations:** 1 Department of Medical, Health & Sports Engineering, MCI, 6020 Innsbruck, Austria; 2 Department of Mechatronics, MCI, 6020 Innsbruck, Austria

**Keywords:** American football, action recognition, convolutional neural network, long short term memory, machine learning, catch training

## Abstract

In order to train receivers in American football in a targeted and individual manner, the strengths and weaknesses of the athletes must be evaluated precisely. As human resources are limited, it is beneficial to do it in an automated way. Automated passing machines are already given, therefore the motivation is to design a computer-based system that records and automatically evaluates the athlete’s catch attempts. The most fundamental evaluation would be whether the athlete has caught the pass successfully or not. An experiment was carried out to gain data about catch attempts that potentially contain information about the outcome of such. The experiment used a fully automated passing machine which can release passes on command. After a pass was released, an audio and a video sequence of the specific catch attempt was recorded. For this purpose, an audio-visual recording system was developed which was integrated into the passing machine. This system is used to create an audio and video dataset in the amount of 2276 recorded catch attempts. A Convolutional Neural Network (CNN) is used for feature extraction with downstream Long Short-Term Memory (LSTM) to classify the video data. Classification of the audio data is performed using a one-dimensional CNN. With the chosen neural network architecture, an accuracy of 92.19% was achieved in detecting whether a pass had been caught or not. The feasibility for automatic classification of catch attempts during automated catch training is confirmed with this result.

## 1. Introduction

Sports have become more data driven in recent years. In competitive and professional sports, all athletes are monitored in nearly every game and, if possible, also during training. The monitoring provides data that can be analysed to further improve the performance of individual athletes or the team, but it can also deliver information about opposition teams, their tactics and strategy, strength and weaknesses, etc. [1,2,3,4,5]. As the amount of available data is too large to be processed efficiently by coaches and analysts, the state of the art in the analysis of such data comprises a mixture of computer-aided and human analysis and evaluation [2,5,6,7,8]. The computer-aided part of the analysis is mostly based on modern algorithms, e.g., methods of machine learning [9,10,11,12,13], though before any analysis can be carried out, the data has to be gathered. Hence, some sort of monitoring device or sensor is needed.

The digi sporting consortium has published an overview of electronic performance and tracking systems (EPTS) under [14], which reflects the state of the art, how monitoring of athletes is achieved in many sports, such as running, soccer and rugby. The major information that should be monitored is highly dependent on the sport. Therefore, a wide range of EPTS are used throughout various sports. Nevertheless, they rely on mostly the same measurement methods to gain the data.

In many sports, the position of an athlete is a highly relevant information, so the major methods to determine an athletes position are either optically based or inertial measurement unit (IMU) based. In some cases, the IMU-based method is extended and combined with some global navigation satellite systems (GNSS), depending on the sport. Saramento et al. reviewed the most common methods for match analysis in soccer in many parts of the world, which relied mostly on video data [4]. At the same time, companies such as Statsports provide products reliant on IMU and GNSS data to determine the performance in soccer and other sports [15,16]. Positions and their change over time were also measured optically by [17,18,19] in their studies throughout several sports. In the context of American football, the position information is also relevant, but there are other metrics relevant from a positional perspective. A more detailed performance analysis, with respect to the position of e.g., a wide receiver, would also include the outcome of a catch attempt.

Pass-receiving athletes are monitored throughout the season of the NFL and NCAA leading to statistics such as the number of receptions, the catch rate or similar statistics [20]. Some more detailed statistics might be the defense-adjusted yards above replacement or the defense-adjusted value over average metric [21]. Unfortunately, there are some major problems with the given data. All mentioned statistics about catching are derived from various actions and outcomes during games in a season. By definition, this statistic excludes a major part of each team, including the practice squad in NFL teams or any athlete without game time. Furthermore, athletes with a relatively low amount of minutes during a game do not have enough opportunities to catch a pass. Therefore, no meaningful analysis of their performance can be derived. In leagues that do not have such statistics, due to the lack of someone to gathers and processes the data, no analysis can be accomplished at all. This means that for the majority of athletes in American football no statistic or performance metric exists, which reflects the catching performance.

This major drawback can be resolved by introducing a system that can gather data from exercises during regular training in American football. By gaining data there, the availability of such data increases drastically. For that reason, Hollaus et al. introduced a system that can distinguish between a successful and an unsuccessful catch attempt [22] that is applicable in regular training. The system was based on IMUs along with a machine learning algorithm, that classifies catch attempts as catch or drop. Several disadvantages go along with the system such as mistriggering, catch attempts might not be recognized as such and the need that all pass receivers must wear two wearables on their wrists that might hinder them in their catching motion. Another possible way to gain information about a catch attempt in training would be based on an IMU in the American football ball. A similar approach is currently taken in sports such as soccer, cricket and recently also American football [23,24,25]. The major drawback there is that it is not very suitable for a regular catch training routine including many athletes, as different balls that contain the IMU would be required. Additionally, it would be necessary to pair every athlete to one ball to guarantee that all catch attempts are performed by the same athlete and enable an individual analysis. By reviewing all potential methods to classify a catch attempt, it became clear that all of the mentioned methods and the given measurement systems have benefits and drawbacks concerning at least one of the following features: accuracy, precision, mobility, robustness, etc. Based on the authors’ experience, a major requirement for any system in sport is that any recording system must not hinder or restrict the athletes in their regular training activities.

Due to this fact, it is investigated whether it is possible to classify audio and video recordings of American football catch training. As the necessary camera and microphone would not be placed on the athletes, these systems do not hinder them in their catch attempt and would represent a major improvement over the original IMU system. In contrast to the approach with an IMU in the ball, a central audio and video recording system would be scalable and independent of the number of athletes participating in the training. Therefore, it is potentially more cost efficient. Additionally, it does not wear out as an American football ball would. Nevertheless, some drawbacks also exist for an audio and video recording system. One of them would be that the information, which can be generated from such a system, only covers the outcome of a catch attempt. It would be much better if the outcome of a catch attempt can be related to a specific catch scenario (e.g., catch a pass over the shoulder during a deep run down the field or catch a quickly thrown screen pass in front of the receivers chest or catch an nearly underthrown pass). If a human quarterback should throw the passes with high precision and accuracy within such a scenario, major limitations to the catch training would be given. The number of passes is limited by the strength of the quarterback, as well as accuracy and precision, which tend to decrease with fatigue. Therefore, a human quarterback is not the ideal solution to the given problem. If the systems should also be able to set a scenario for the catch attempt, it is mandatory to control the pass to the athlete via a passing machine. At the same time, it enables a more detailed analysis of the catch attempt under specific scenarios. For this paper, the recording system should be seen as a part of the passing machine, not as a stand alone system.

As the recording system was integrated into a passing machine, it became necessary to further define, which passing machine should be used. Passing machines have existed in American football for several decades [26,27]. In the last few years passing machines became more automized and controllable. A company named Monarc introduced a fully automated passing machine called the Seeker [28]. All the given systems have no open interface for integrating external hardware. Therefore the passing machine, which was designed by Hollaus et al. [29,30], is the only one that enables the development of the catch recognition system, based on audio and video recordings. Still, the passing machine and the recording system only enable a profound analysis of the catching abilities of receiving athletes but an algorithm that analyzes is still missing.

The field of action recognition in sports heavily relies on algorithms based on machine learning [31]. As the data are manifold in the given circumstance, there is the need of a so-called time series classification algorithm on a signal basis for the audio data, but there is an additional need to process a series of images containing the catch attempt of the respective athlete. In the classification of time series data, Fazle et al. showed excellent results when using a Long Short-Term Memory Fully Convolutional Network (LSTM-FCN) [32]. Fazle et al. also adopted this concept and could further improve the accuracy of the classification [33]. Ref. [34] shows an overview of common methods for classifying time series data. The classification of video or image series data is, due to the high computational complexity, challenging. However, recent research results show very good results in the area of activity recognition [35]. Tran et al. showed a method for classifying videos using Channel-Separated Convolutional Network (CSN). Very good results in activity recognition are shown by Donahue et al. A Long-Term Reccurent Convolutional Network (LRCN) is used as the model architecture [36]. In the field of sports, machine learning has been used to classify sports [37]. There are also studies on human activity recognition, gait analysis or human pose estimation [38,39,40,41,42,43]. In human pose estimation, the classification is performed mainly using data from image capture [44]. Based on the literature and state of the art in close fields, it can be imagined that it is possible to analyze catch attempts in an automized way by applying the mentioned methods on audio and video data of automated catch training.

Therefore, the main goal of this paper is to provide a system and an algorithm that allows the automated analysis of a catch attempt in American football based on audio and video data. The analysis should identify a catch or a drop with reasonable accuracy. Based on [22] the accuracy should be at least close to or better than 93%.

## 2. Material and Methods

In this section the used methods are given in chronological order. First, the audiovisual recording device and its integration into the given passing machine is outlined. The data acquisition phase is shown secondly. Next, the preprocessing algorithms, including labeling, are explained in detail. This section closes with the development of neural networks for audio and video classification.

### 2.1. Audiovisual Recording System

**Figure 1:**
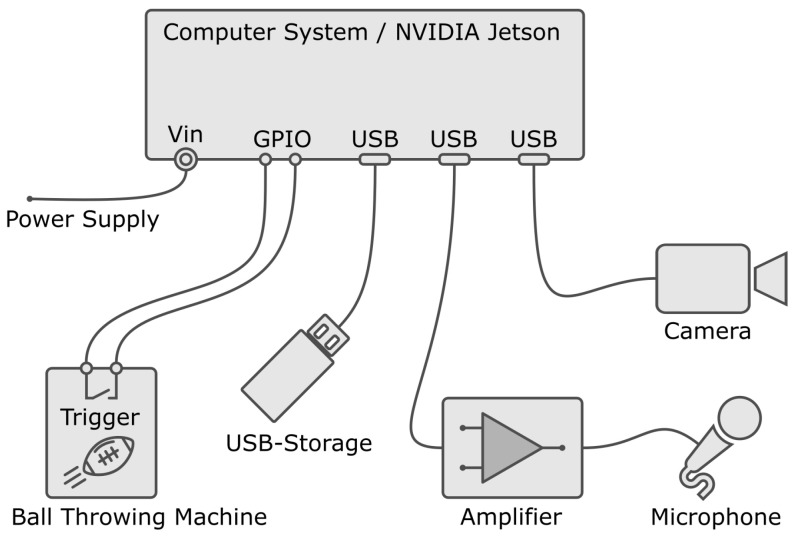
Blockdiagram of the audivisual recording system.

To train the neural networks, a recording system is needed for data acquisition. This system should independently record and store an audio and video sequence of an athlete during a catch attempt via an external trigger. The recording system is integrated into the passing machine [29,30], which triggers the start of the recording. A camera and a microphone with a directional pattern are mounted on the ball-throwing machine. After triggering, an audio sequence and an independent video sequence are recorded. An overview of the recording system is shown in the system topology in Figure 1. The central point of the system is the computer system. The camera, the microphone with the amplifier, the external USB memory storage and the trigger contact of the passing machine are connected to it.

For the video recording, it was necessary to choose a camera that fits the requirements of the experiment. The requirements were defined as follows. The receiving athlete may attempt to catch the pass in a distance between 10 m to 50 m away from the passing machine. For the whole range, it is necessary to record the entire body of the athlete during catching. As various scenarios for the catch attempts are considered, including a catch attempt while running or jumping, it is necessary to have at least a field of view of 4 m. This requirement can be met with an opening angle of 22.7° and a sensor size of at least 1/2. Therefore the optics were chosen according to given need with the lens Edmund Optics 16 mm f/2.8 Ci-Series. From a camera perspective, it was important to determine the minimum frame rate, recording time and resolution. As the catch attempt starts when the pass is thrown and ends when the pass is successfully caught or not, the recording time was considered to be several seconds. Within the recorded seconds, the information about the outcome of a catch attempt should be easily visible within the recorded frames. Considering a frame rate of 1 fps and a recording time of 5 s, five recorded frames would be the outcome, with images before and after the potential catch happened. The authors assumed that a classification is possible based on a few frames that show the catching motion before and after the respective catch happens. Nevertheless, a camera was chosen that can record a frame rate of 60 fps, to have a better coverage and be able to find the minimum frame rate based on an experimental approach, not on an assumption. The camera was configured with a resolution of 640 × 512 pixels, since machine learning algorithms that use images as an input often only need even lower resolutions than 640 × 512 pixels [45].

A microphone should be used to record the characteristic catching sounds of the football. Since the sounds should also be recorded up to a distance of 50 m, a microphone with directional characteristic needs to be chosen. This is also beneficial for the damping of any external noise that comes from other sound sources in the surroundings. Most of the directional microphones need so-called phantom power along with an amplifier to have a well established recording quality. Based on the given requirements, many possible microphone setups would be the outcome. The authors chose Rode NTG-2, but also state that many other setups would be possible. Since the microphone requires external 48 V phantom power, the recommended audio amplifier Steinberg UR22mk2 is used. The operation was performed via a USB 2.0 interface. The audio signal was recorded with a sampling rate of 48 kHz, which is sufficient for the needs of the experiment.

A central computer system is used to record the audio and video sequence. In the scientific community, different central computer systems are accepted, especially within image processing [46]. The system which was chosen for the experiment is a powerful System-on-Module (SOM) from *NVIDIA* called *NVIDIA JETSON*. It features a dedicated *NVIDIA Maxwell* graphics processor with 128 cores, a *quad-core ARM A57* processor and 4 GB *LPDDR4* RAM. The power supply is provided by a 5 V/ 4 A plug-in power supply. The operating system is loaded onto an SD card. To reduce the write access to the SD card, the audio and video sequences are stored on an external data carrier (USB3.0/ 128 GB) as can be seen as USB storage in Figure 1. A metal case was used to protect the system from damage. The general purpose input/output (GPIO) to trigger recording is routed to a housing connector.

### 2.2. Experimental Setup and Data Acquisition

The data that are necessary to train the networks were gathered in an experiment. The experimental setup always consisted of a passing machine [30] which also carried the recording system. The recording system was connected to rotate horizontally according to the azimuth of the launch unit of the passing machine. Therefore, the orientation of the microphone and camera is always the same as the horizontal orientation of the launch unit of the passing machine. The passing machine was instructed to run a pass routine by pressing a button on the machine. The pass routine starts with a short acoustic warning signal. This warns the receiving athletes so they are aware that a pass will be released just after the warning signal ends. This also triggers the audio and video recording so the catch attempt is covered from pass release to a few seconds after the end of a catch attempt was made. The recordings were then stored on the external USB-Storage according to Figure 1.

All participants were only instructed to attempt to catch the pass and try various catch motions (e.g., faced toward the machine, while running, over-the-shoulder catches, or similar). There were no further instructions for the catching process. The experiment was designed to have as much variability as reasonably possible. Therefore, the experiment was carried out with a total of thirteen different athletes. All the players were amateurs. Most players have never caught an American football ball before and are not entrusted with catching techniques. In this data, it is important to note that four of the thirteen players with percentages of 67.57% are included in the dataset.

The experimental design was approved by the ethics committee of the MCI and all participants have signed a declaration of consent. To enhance variability within the dataset, the data recording was performed at two different locations, the auditorium at MCI and a parking lot. The recordings at the MCI auditorium site were made indoors in the building and recordings at the parking lot were made outdoors. At each location, passes are taken with different background types. This should lead to a higher robustness of the neural network with respect to a change in the environmental parameters. A total of five different background types were chosen. In the outdoor area, the background types shrubbery, shrubbery & wall and building are included in the dataset. In the indoor environment, two background types are recorded, glass doors and a light background. The recorded dataset, in a volume of 2276 passes, forms the basis for training the neural networks.

### 2.3. Labeling and Data Processing

The labeling of the data is indispensable for the training of neural networks. Therefore, the labeling of the individual data is performed by the file name and consists of several parts. The name of the recording system, information about key frames of the video, the class and subclass, the recording location and the player are stored. Audio and video recordings are stored separately. To still be able to assign the data to each other, they are labeled exactly the same—except for the file extension. In addition to the two main classes Catch and Drop, subclasses are also formed. These subclasses contain the movement pattern of the athlete during the catch the pass. The subclasses Jump, One-handed, Run and Stand are formed. The catch types are not used for training the neural networks. However, the subclasses can be used to analyze the dataset and for more future work. An important metric for training is the ratio of caught passes (class: Catch) and uncaught passes (class: Drop). This ratio can be seen in Table 1.

The Drop class has a significantly smaller share of the total amount of data with 669 recorded passes. When training the neural network, this can lead to the fact that data belonging to the class Drop are not classified with the same high accuracy as data of the class Catch [47].

Before the data ar fed to the neural network, it was processed in three steps. First, the data were preprocessed, then duplicated, and finally stored in a specific file format.

When the video data were processed, the individual recordings are converted into a four-dimensional matrix of the form 209×512×640×3. To train the neural network with different datasets, the videos are scaled differently and their frame counts are varied. The *OpenCV* scaling function is used to scale down the video. The frames are reduced to four different sizes 100×100, 150×150, 200×200 and 250×250 pixels. In addition to reducing the size of the frames, the number of frames in the video is reduced. The reduction is performed using the number of the key frame at which the player touches the ball. Based on this time point, only frames that are 0.5 s before and 1.5 s after this time point are further used. Thus, the time range is 2 s. Three time intervals 0.5 s, 0.2 s, and 0.1 s are defined between frames. The resulting videos have frame counts of 5, 12, and 21. After data reduction, the videos are normalized to the range of values [0, 1]. The audio data, similar to the video data, are trimmed, interpolated and stored as a matrix. The audio data, like the video data, are trimmed to a period of 2 s. 0.5 s before and 1.5 s after the player touches the ball are used for the sequence. The data are reduced to a size of 50,000 × 1 via interpolation. Analogous to the video data, the audio data are also normalized. The normalization is performed to the range of values [−1, 1].

**Figure 2:**
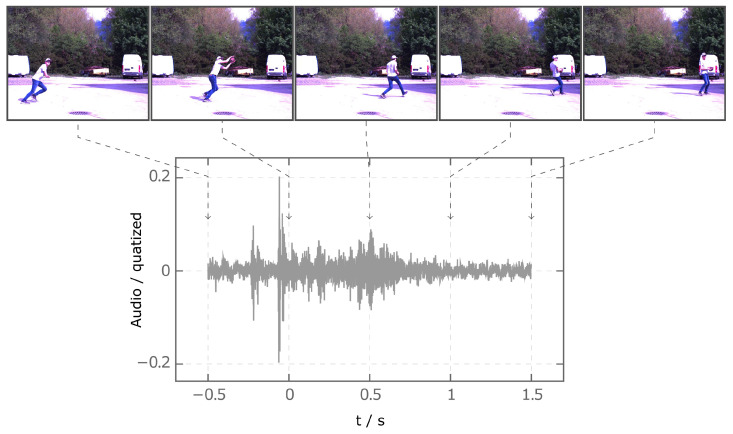
Processed audio and video data of class Catch in a synchronized fashion. Every extracted frame is connected to the specific time stamp in the audio data.

Figure 2 shows the normalized audio and video data of class Catch. Here, the video has the format 5×200×200×3. The audio and video data are time-synchronized. For every trimmed sequence, the recorded time is given a unique offset such that the time is 0 s when the athlete first touches the ball.

To increase the accuracy and robustness of the neural network, the data are multiplied. Too little data can cause the neural network to generalize poorly and thus produce poor results on unknown data [48]. Duplication of the data is applied to the video data and to the audio data [48]. The video data are duplicated using four different methods [49]. The horizontal mirroring and cropping of the image, the addition of noise, and histogram equalization. Duplicating the audio data is also performed with four methods. Two methods, adding noise and adjusting gain, change the amplitude of the signal. Two other methods make changes to the time course of the signal. Shifting and stretching or compressing the audio signal. Especially when augmenting input data, overfitting of the network must be cautiously avoided. Therefore, all network performances are judged using test data as shown in the Results and Discussion section. Since the processing of the data takes a lot of time and the dataset cannot be completely loaded into the working memory, it must be cached on the hard disk and loaded sequentially for the training of the neural network. The *Tensorflow* proprietary *TFRecord* format is used to store the data. This simple binary format is used for storing large datasets and is optimized for *Tensorflow* [50]. In the initial development phase tensorflow version 2.2.0 was used. In addition to conversion, data are split before saving. Splitting is performed into a partial dataset for training (64.9%), validation (12.2%), testing (22.9%). The splitting of the dataset is achieved with the function *StratifiedShuffleSplit*, of the *scikit-learn* library. This function has the advantage that the splitting of the classes is evenly distributed in all splits.

### 2.4. Development of Neural Networks

The recordings contained in the dataset, consisting of audio and video data, are used to train neural network models. These models are designed to perform binary classification. The audio recordings consist of univariate time series data. The video data, on the other hand, is composed of images in a specific time sequence. Several models are implemented for classification due to the different data types. The optimization of the model structure and hyper-parameters of all models is performed empirically. The optimization process is performed in two stages. The optimization of the models is achieved using only the training dataset of the raw data. No data duplication is used to reduce the computation time. In the first stage the model structure is optimized and with the next stage a fine optimization of the hyper-parameters was performed.

#### 2.4.1. Classification Based on Audio Data

**Figure 3:**
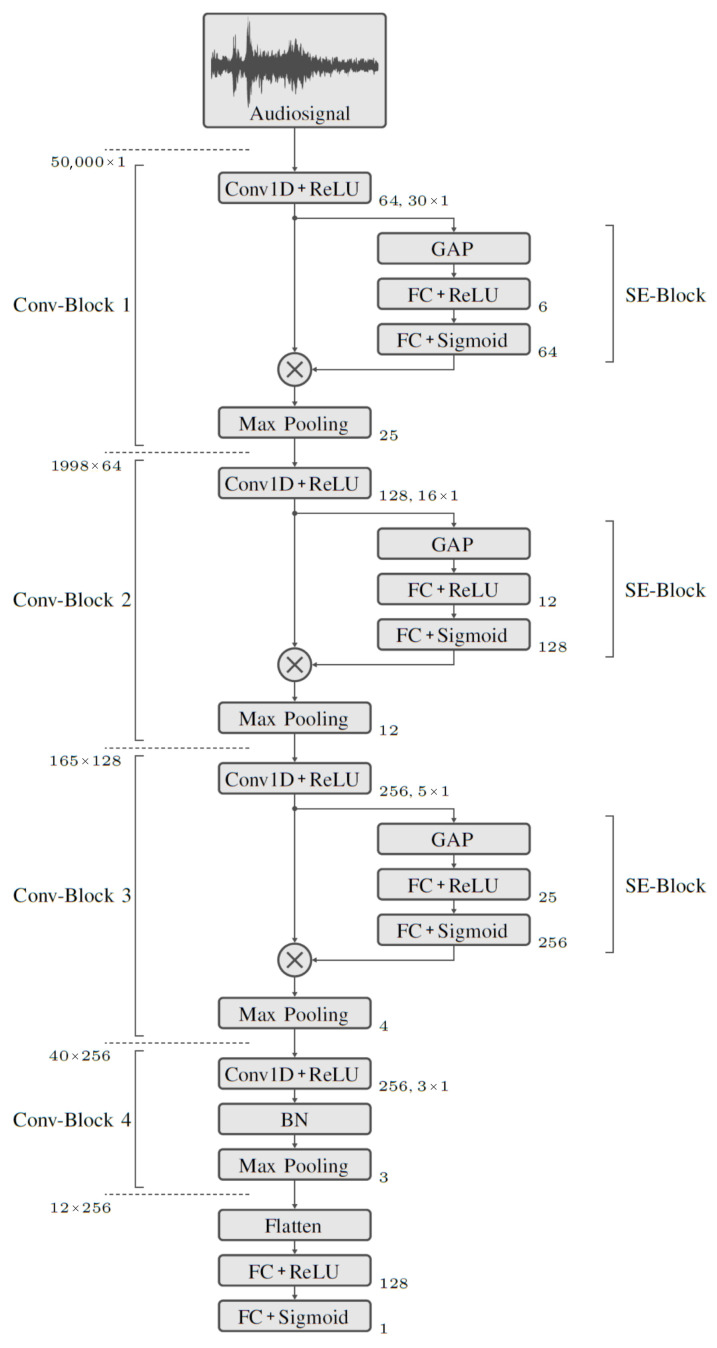
Network structure of the audio model with the applied operations. To the right of the operations, their set parameters can be seen, such as the number of neurons, filters and filter sizes. The size of the tensor, at the model input and at the output of each of the convolutional blocks, is shown on the left side.

The classification whether a record belongs to the class Drop or to the class Catch is performed with a first model purely based on the audio recordings. For classification, a model architecture with several Convolutional layers connected in series and a fully meshed output layer is used. An overview of the network structure of this model is shown in Figure 3. The input to the model is the audio signal in the form of a one-dimensional tensor with 50,000 elements. This is followed by four convolutional blocks, *Conv-Block 1* through *Conv-Block 4*. These are used to extract signal features. The final classification is performed using Fully Connected (FC) layers. The special feature of *Conv-Block 1* to *Conv-Block 3* is the downstream Squeeze and Excitation (SE) block. Ref. [51] demonstrates that SE blocks provide significant performance improvements with little additional computational overhead. The model is trained with the training dataset. Since the classification is binary, the *Binary Crossentropy* loss function is chosen. As an optimization function, for updating the weights during training, Adam [52] is used. The batch size is set to 5. During training, the learning rate is adjusted after each epoch. The best results are obtained with an initially higher learning rate of 1 × 10^−5^, which decreases linearly over 20 epochs to 8 × 10^−6^. This is kept stable over 40 epochs and then exponentially reduced to 2 × 10^−6^.

#### 2.4.2. Classification Based on Video Data

**Figure 4:**
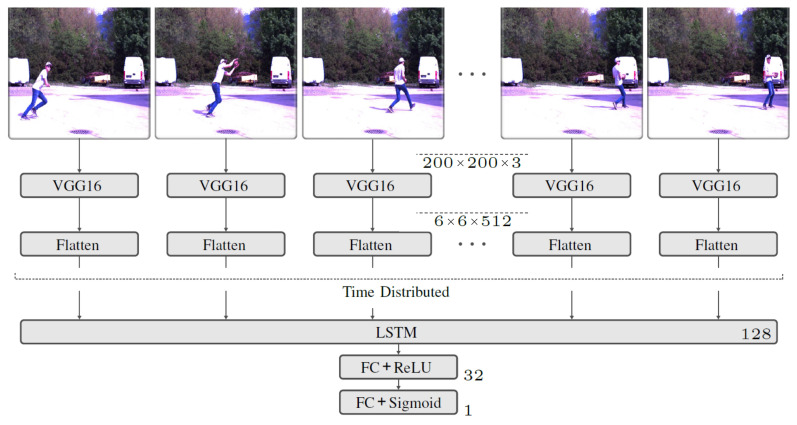
Network structure of the video model with the applied operations. To the right of the operations, their set parameters can be seen, such as the number of neurons, filters and filter sizes. The size of the tensor, at the model input and at the output of the VGG16 network, is shown in the middle.

A CNN is used to extract the features contained in the individual images. However, since training a CNN to classify image data requires a very large dataset to achieve high accuracy, pre-trained network is used for feature extraction. Specifically, the VGG16 [45] Network is embedded. This is a very compact mesh with relatively few parameters. The network was developed to classify images and has been used with 1.28 × 10^6^ Images trained on 1000 classes. It gives very good results on the *ImageNet* dataset. Since a video consists of multiple frames, feature extraction is applied to all frames separately using *TimeDistributed* function. The output of the feature extraction has an additional dimension that describes the temporal flow of the extracted features. An LSTM is used to account for temporal dependencies between the extracted features. The network structure of the implemented model is shown in Figure 4.

The model input is a tensor of the form number of frames×200×200×3. An image dimension of 200×200×3 is chosen because the VGG16 network was developed with images of dimension 224×224×3 and is optimized for this purpose. Finally, the feature extraction contains a *flatten* operation to suitably restructure the tensor for use with the LSTM. To learn the temporal dependencies between the extracted features, an LSTM with 128 cells is used. The final block, which also contains the final output layer for classification, is formed by FC layers, similar to the audio model. The training dataset is used to train the model. Since the classification is binary, *Binary Crossentropy* is chosen as the loss function. Adam is used as the optimization function. A batch size of 6 is used. During training, the learning rate is adapted after each epoch. The best results are obtained with an initial learning rate of 2 × 10^−5^. This is exponentially reduced from epoch 2 to 8 × 10^−6^.

#### 2.4.3. Classification Based on Audio and Video Data

**Figure 5:**
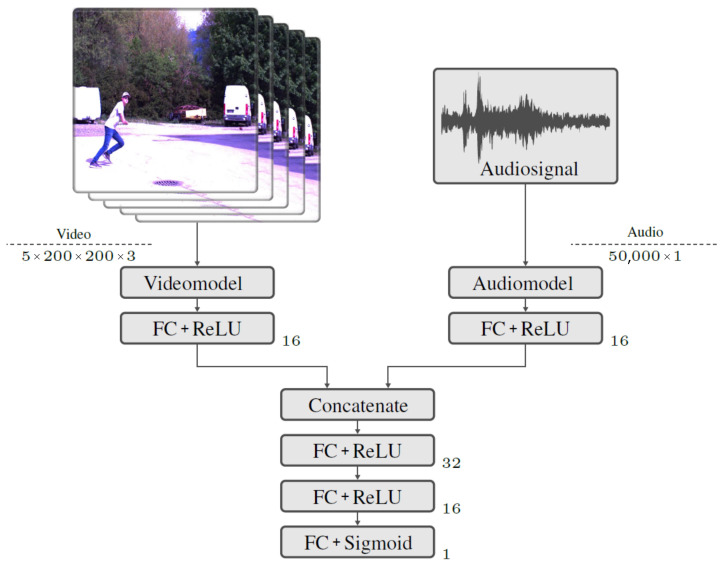
Network structure of the audio/video model with the applied operations. To the right of the operations, their set parameters can be seen, such as the number of neurons, filters and filter sizes. The size of the input tensor is shown per data type.

The pre-trained models for audio and video classification were integrated and linked into a third model. This should lead to higher accuracy in the classification of the data, since the entire dataset with the audio and video source is used for the prediction. The network structure shown in Figure 5 provides an overview of the model. The video data are processed using a model branch with the video classification model already trained. The input tensor of the video data has the same size as the input tensor of the video classification model.

The second network’s input is the audio data. The input processing is performed with the already trained network of audio classification. The input tensor of the audio data has the same dimension and size as the input tensor of the audio model. The last layers, which are used for classification, are removed in both models. Instead, a fully connected (FC) layer with a Rectified Linear Unit (ReLU) activation function is used. The two model branches for processing the audio and video data are linked using the *Concatenate* function. Two FC layers follow. The output layer for binary classification is an FC layer with a sigmoid activation function. The training of the model is performed with the audio and video training datasets. *Binary Crossentropy* is chosen as the loss function since the classification is binary. A batch size of 3 is used. During training, the learning rate is adjusted after an epoch change. The highest accuracy on the test dataset is obtained with an initial learning rate of 1 × 10^−5^. The learning rate is exponentially reduced to 5 × 10^−6^ from epoch 2.

## 3. Results

As a result, all networks are evaluated on their accuracies and robustness. To test whether the training dataset used impacts the classification result, the models are trained with different training datasets. For this purpose, different combinations of training datasets of the raw data are tested with duplicated data. The achieved accuracy in the classification of the test dataset serves as a comparison value.

### 3.1. Performance of the Audio Network

The accuracies achieved by the audio network using different training datasets are shown in ascending order in Table 2. Training the network with a combination of raw data and stretched/compressed data in time causes a significant degradation in accuracy compared to the result of the raw data alone. The combination of raw data and data where the audio signal is amplified shows no significant improvement to the result of the raw data.

On the other hand, two combinations of datasets show a significant improvement in the achieved accuracy. The combination of raw data with shifted signals and the combination of raw data and the signal with noise both produce an increase in accuracy. Finally, the audio model is trained with a combination of the raw dataset and the datasets with shifted and noisy audio signal. The result can be increased again with this combination.

The evaluation of the classification, such as accuracy, hit ratio and F1-measure shows very low values for the class Drop. The class Catch, on the other hand, shows better values in the classification. The results of these metrics are shown in Table 3.

### 3.2. Performance of the Video Network

The accuracies achieved by the video model on different training datasets are shown in ascending order in Table 4. Training the model with the raw data only gives the worst results. No improvement in accuracy is shown by combining raw data and cropping. Good results are obtained with dataset combinations of raw data with mirrors, noise or histogram. Using all datasets when training the model shows no advantage over a simple dataset combination. However, the highest accuracy can be achieved with the dataset combination of raw data with mirrors, noise and histogram.

This dataset combination is used for further investigation in training the model. The effect of the size of the frames of the video on the achieved accuracy is tested. The model is trained using datasets with four different frame sizes. Table 5 shows the results of classification on the test dataset using different video configurations. The results show an increase in model accuracy with increasing image size up to an image size of 200×200 pixels. Higher resolution images do not produce better results with this model.

Another test is performed. In this one, it is tested whether the number of frames of the video used for training has an impact on the model accuracy obtained. The model is trained with three different datasets. Videos with 5, 12 and 21 frames are used. The results of classification on the test dataset can be seen in Table 6. Increasing the frames used per video also improves the accuracy of classification to some extent. The maximum accuracy can be achieved with the dataset where 12 images are used. Increasing the images per video to 21 does not improve the result. The major drawback of increasing the number of images, is the huge increase in computational cost for training and classification.

The model evaluation is performed using the key figures for the individual classes, such as accuracy, hit rate and F1 measure. These can be seen in Table 7 and show, compared to the audio classification, significantly higher accuracy for the classification of the class Drop. The class Catch is classified, compared to the audio model, with a slightly higher accuracy.

### 3.3. Performance of the Combined Network

The accuracies achieved by the combined network, using different training datasets, are shown in Table 8. Training the model with a combination of raw data and augmented data leads to worse results in most cases. Only a slight improvement in accuracy is shown by the combination of raw data and augmented data by using methods such as shifting and amplification for audio and mirroring and histogram equalization for video data. This dataset combination has the main advantage that the amount of data and therefore the computational effort is low.

The results show a different behavior when using augmented data as input for the combined network when compared to the audio or video model. The studies of the audio model and also the video model show an improvement in accuracy when using augmented data for training the models. The audio/video model, on the other hand, shows a decrease in accuracy in most cases, with one exception.

Using the metrics for each class, such as accuracy, hit ratio, and F1 measure, the model is evaluated. The classification of the class Drop can be further improved by an absolute value of 6%. The metrics can be seen in Table 9.

## 4. Discussion

The results showed that, when using only the audio network it is not possible to classify the data reliably. This model cannot be used as an independent system due to low accuracy in the classification. The information content of the recorded audio data was too low to achieve a higher accuracy. Analyses of the dataset show that in some cases no audio signal of the ball hitting the player is recorded. This happens when the player is too far away from the microphone or when too much ambient noise overlays the recording. Recordings in a sports hall would show an improvement. The ambient noise can be minimized and the quality of the audio recording can be increased.

The evaluation of the video network shows that classification of video data is possible. The class Catch can be determined very well. The hit rate for the class Drop is still not sufficient for a reliable classification of the data with 79%. However, it is important to keep in mind that the size of the dataset is very small. With a larger dataset, higher accuracy may be achieved. An increase in accuracy when using a larger dataset can already be observed when optimizing the model.

The combined use of the audio and video network shows a strong improvement in the classification of catch attempts. In comparison with the IMU approach in [22] the performance is close. An overall accuracy on the test dataset of 92.19% is not yet sufficient for the use of the model in a fully automatic training system. However, this result demonstrates the feasibility of such a system.

There are also limitations of the system regarding its performance outside the given scenarios of the experiment. In the experiment, only amateurs participated in two different locations. This means, that other backgrounds, other athletes wearing other sportswear that have other individual catching skills, might lead to worse accuracy. This issue can only be solved by creating more versatile data in many different places with many different athletes that have a wide range of catching skill level. Nevertheless, the initial goal was to show that catch recognition can be achieved in American football using machine learning based on audio and video data. This goal was achieved.

Another major limitation was the number of athletes within the area of sight of the camera. In a training scenario, the background might not be as static as it was in the given scenarios during the experiment. On a pitch, there might be more dynamic backgrounds, which could lead to a worse performance. As no dynamic backgrounds were part of the dataset, the network did not learn how to deal with them properly. As already mentioned, the amount of data and the versatility of it is a major limitation of the given system. Though, within the given constraints of the dataset the outcome is acceptable.

## 5. Conclusions

As part of the paper, an audiovisual recording system was developed. This offers the possibility to record a football athlete during catch training. Together with an automatic ball-throwing machine, a comprehensive dataset was created. The dataset consists of audio and video recordings with a dataset of 2276 recorded catch attempts. This was preprocessed and duplicated by applying different augmentation methods. Three neural networks were developed and optimized to classify the data. An evaluation of the three models showed that the classification of the data was possible. From the individual model tests, it was found that the audio model achieved the worst result in the classification. The model for classifying the video data achieved good accuracy. The best performance was achieved with a combination of the audio and video network. It was shown that the classification of audio and video data is possible. The achieved accuracies of classification of 92.19% confirm this study. Through the research accomplished in this work, a system for automatic training of athletes can be developed. With such a system, it is especially possible to gain new knowledge in the field of training athletes and developing training methods with fully automatic systems. In comparison to the IMU-based system, the performance of the combined network is slightly worse. Nevertheless, the major advantage of the combined audio video approach is that there is no need to put wearables on the athlete. Therefore, the training routine of receivers does not change, which most likely would result in better acceptance of the system by coaches and athletes.

According to the authors, further research should focus on identifying the subclasses of the catch motion, such as Jump, One-handed, etc. Another question that remains unanswered is whether the improvement in audio quality allows for reliable audio-only classification. Likewise, the classification using only video data could have interesting applications in broadcast recordings of sports matches.

Another area that was not researched yet is the automated training and the development of a performance index according to the data, recorded during automated training. It can be imagined that an automated passing machine, that throws a pass accurately, can throw passes athletes in a randomized way (e.g., 100 passes in total within the area of reach of an athlete, 25 passes in each quadrant from seen from the athletes chest). Based on the information if the athlete caught a pass or not, a performance index could be derived, according to the quadrants. This approach could be extended to any other catching scenario in American football catch training as the information is surely useful for coaches, scouting, athletes, fans, etc.

## Figures and Tables

**Table 1:** Catch Attempts per Class.

Class	Amount	Ratio in %
Catch	1607	70.61
Drop	669	29.39

**Table 2:** Achieved accuracies of audio classification on the test dataset, using different combinations of the training dataset.

Training Dataset					Accuracy
Raw Data	Shift	Amplify	Noise	Stretch/Compress	in %
Yes	-	-	-	Yes	76.88
Yes	-	-	-	-	78.18
Yes	-	Yes	-	-	78.70
Yes	Yes	-	-	-	80.52
Yes	-	-	Yes	-	80.52
Yes	Yes	-	Yes	-	81.04

**Table 3:** Model assessment with the test dataset in audio classification.

Class	Accuracy	Hit Rate	F1 Measure	Number of Catch Attempts
	in %	in %	in %	
Drop	69	62	65	111
Catch	85	89	87	274

**Table 4:** Achieved accuracies of video classification on the test dataset, using different combinations of the training dataset.

Training Record: 5×200×200×3					Accuracy
Raw Data	Mirror	Crop	Noise	Histogram	in %
Yes	-	-	-	-	83.07
Yes	-	Yes	-	-	83.33
Yes	-	-	Yes	-	85.68
Yes	Yes	Yes	Yes	Yes	85.68
Yes	-	-	-	Yes	85.94
Yes	Yes	-	-	-	86.46
Yes	Yes	-	Yes	Yes	86.98

**Table 5:** Achieved accuracies of video classification on the test dataset, for datasets with different image sizes. Raw, mirror, noise and histogram datasets are used respectively.

Training Dataset	Accuracy in %
5×100×100×3	82.03
5×150×150×3	85.45
5×200×200×3	86.98
5×250×250×3	86.60

**Table 6:** Achieved accuracies of video classification on the test dataset, for datasets with different number of frames. Raw, mirror, noise and histogram datasets are used respectively.

Training Dataset	Accuracy in %
5×200×200×3	86.98
12×200×200×3	90.36
21×200×200×3	88.80

**Table 7:** Model evaluation with the test dataset in video classification.

Class	Accuracy	Hit Rate	F1 Measure	Number of Data
	in %	in %	in %	
Drop	86	79	83	111
Catch	92	95	93	273

**Table 8:** Achieved accuracies of audio/video classification on the test dataset, using different combinations of the training dataset.

Training Record						Accuracy
Raw Data	Audio:	Shift	Stretch/Compress	Noise	Amplify	in %
	Video:	Mirror	Crop	Noise	Histogram	
Yes		Yes	-	-	-	86.72
Yes		-	Yes	-	-	88.80
Yes		Yes	Yes	Yes	Yes	90.36
Yes		-	-	Yes	-	90.63
Yes		-	-	Yes	Yes	91.14
Yes		-	-	-	Yes	91.15
Yes		-	-	-	-	91.92
Yes		Yes	-	-	Yes	92.19

**Table 9:** Model assessment with the test dataset in audio/video classification.

Class	Accuracy	Hit Rate	F1 Measure	Number of Data
	in %	in %	in %	
Drop	88	85	86	111
Catch	94	95	95	273

## Data Availability

The data presented in this study is available on request from the corresponding author.

## Abbreviations

The following abbreviations are used in this manuscript:

MCI	Management Center Innsbruck
IMU	inertial measurement unit
ICA	independent component analysis
etc.	et cetera
ADC	analog digital converter
e.g.	exempli gratia
CNN	convolutional neural network
LSTM	long short time memory
ReLu	rectified linear unit
ML	machine learning
FC	fully connected
